# Gastrointestinal Helminths and Ectoparasites in the Stray Cats (Felidae: *Felis catus*) of Ahar Municipality, Northwestern Iran

**Published:** 2017

**Authors:** Mohammad YAKHCHALI, Nasser HAJIPOUR, Reza MALEKZADEH-VIAYEH, Bijan ESMAEILNEJAD, Taher NEMATI-HARAVANI, Mohammad FATHOLLAHZADEH, Rasool JAFARI

**Affiliations:** 1. Parasitology Division, Dept. of Pathobiology, Faculty of Veterinary Medicine, Nazlu Campus, Urmia University, Urmia, Iran; 2. Parasitology Division, Dept. of Pathobiology, Faculty of Veterinary Medicine, University of Tabriz, Tabriz, Iran; 3. Lake Urmia Research Institute, Urmia University, Urmia, Iran; 4. Dept. of Medical Parasitology, Faculty of Medicine, Tabriz University of Medical Sciences, Tabriz, Iran; 5. Dept. of Parasitology and Mycology, School of Medicine, Isfahan University of Medical Sciences, Isfahan, Iran

**Keywords:** Helminths, Ectoparasites, Stray cat, Iran

## Abstract

**Background:**

The stray cats are considered as the sources of emerging humans and domestic livestock pathogens and the zoonoses of public health importance. The present study was aimed to elucidate intestinal helminth infections and infestation with ectoparasites of the stray cats of Ahar City, northwestern Iran.

**Methods:**

Totally, 51 stray cats were randomly trapped from different parts of the city between Mar and Nov 2013. The cats were assessed for ectoparasites by hair brushing, skin scraping, acetate tape preparation and othic swabs. They were euthanized and inspected for helminths infection.

**Results:**

Overall prevalence of helminths and flea were 44/51 (86.3%) and 31/51 (60.78%), respectively. The infection rates were significantly different among different age groups (*P*<0.05). Of the 282 isolated helminths, three species of nematodes (*Toxocara cati* (86.3%), *T. leonina* (11.77%), *Ancylostoma tubaeforme* (5.9%)) and four species of cestodes (*Taenia taeniaeformis* (64.7%), *Mesocestoides lineatus* (49.02%), *Dipylidium caninum* (29.41%), *T. hydatigena* (19.6%)) were identified. The predominant infectious helminths in all the infected cats were *T. cati* (86.3% with egg per gram of feces 27.75±9). Of the 270 collected fleas, two species of *Ctenocephalides felis* (80%) and *C. canis* (20%) were notably frequent in the cats aged 2-3-year-old. The average number of fleas per each infected cat was recorded as 5.29, with no incidence of cross-infection.

**Conclusion:**

The results indicated the high rate of helminths infections and flea infestation in the urban stray cats of which *Toxocara cati* and *Ctenocephalides felis* may play important roles as zoonotic agents in the region.

## Introduction

The cats of the family felidae are the main source of a broad spectrum of parasitic diseases in tropical and sub-tropical regions causing health problems, e.g. toxoplasmosis, giardiosis, visceral larva migrans syndrome (VLMS), ocular larva migrans syndrome (OLMS) and hookworm infection, in human and domestic livestock (1–2). The stray cats, *Felis catus*, roaming around human dwellings, streets and public areas, are considered as one of the common transmitters of emerging human and domestic livestock pathogens and zoonoses of public health importance (3).

The environment infects through feces of the felids and causes health problems in humans and domestic livestock with economic losses in Iran (4). Ectoparasites are also common causes of skin diseases as well as vectors of a range of zoonotic diseases in stray cats worldwide. Several studies have been published regarding the distribution and prevalence of ectoparasites infestation in cats (5–6). Accumulation of information about the prevalence of feline parasites is crucial for their control programs. Several studies have been conducted assessing the intestinal helminths in stray cats of Iran (7). However, there still is a lack of information about the prevalence of the parasites infection in stray cats of many regions in the country.

This study was aimed to determine the occurrence and species diversity of intestinal and ectoparasites in stray cats of a region for which no previous data is available.

## Materials and Methods

### Study area

Ahar is located in northwestern Iran (39°5″ N and 47°33″ E) with an area of 3073.93 km^2^ at 1341m above sea level. It is one of the largest cities in the province of East Azerbaijan and known as the main site of agricultural and animal husbandry activities. The maximum and minimum temperatures are 34 °C in summer and −17 °C in winter, with annual rainfall of about 100mm.

### Animals

Totally, 51 stray cats (13.7% females and 86.3% males) were randomly trapped alive from Apr to Dec 2013. The sample size was estimated based on the formula (8). The cats were transferred to the Laboratory of Zoology of Tabriz University, and the data pertaining to their age, sex and body weight were recorded. The age was determined based on eruption of permanent incisor teeth (9). The animals were divided into three groups, namely kitten (less than 1-year-old, n=10), and young (1–2 yr old, n=18) and adult (over 2 yr old, n=23) cats. The examined stray cats sedated by diethyl ether and euthanized with ketamine (100mg kg^−1^, IV) inconsistent with the panel’s recommendation on Euthanasia of the American Veterinary Medical Association.

### Parasites collection and parasitological procedures

#### Helminths

The gastrointestinal tract was tied off at both ends and removed to be inspected for helminths parasites. The parasites were collected and relaxed at 4 °C for 24 h. Nematodes were fixed in 5% ethanol-glycerin and cleared by lactophenol. Cestodes were kept in alcohol-formalin-acetic acid (AFA) solution and stained with Schneider’s acetocarmine staining method. The helminths were identified using the standard keys described by Soulsby (10).

The fresh fecal samples were directly collected from rectum of all the examined stray cats. A part of each sample (3g) was mixed with 42mL tap water. The mixture was subjected to centrifugal sedimentation (1500rpm for 3min) and sucrose floatation methods for isolation of the helminths eggs. The intensity of infection was estimated as number of eggs per gram of the feces (EPG coefficient) (11).

### Ectoparasites

The stray cats were examined for ectoparasites infestation by hair brushing, skin scraping, acetate tape preparation and othic swabs for at least 15 min on the dorsal and ventral trunk (12). Collected ectoparasites were preserved in glass containers by 70% ethanol. The fleas were immersed in 5% potassium hydroxide (KOH) for 15 min. The samples were dehydrated by using serial concentrations of alcohol (50%, 60%, 70%, 80%, 90%, 95% and 100%) for 5 min and then cleared by xylene for 5 min. All the fleas were counted and microscopically identified at 400× as described (13).

### Statistical analysis

The data were analyzed by using non-parametric Chi-square and Fisher exact tests with a confidence interval of 95% (SPSS 16.2, SPSS Inc., and Chicago, IL, USA). Probability of ≤ 0.05 was regarded as significant.

## Results

### Helminths prevalence and species diversity

Data pertaining to prevalence in different age groups and sex are tabulated in Table 1. Overall, 44 out of the 51 examined cats (86.3%) and 29 out of the 51 fecal samples (56.9%) were positive for helminths infection without clinical signs.

**Table 1:** Infection rates of the parasites in different age groups of male and female stray cats from Ahar City, Iran Notes. EPG: egg per gram of feces, F: female, M: male.

**Parasite**	**Age (yr, %)**				**Sex (%)**		**Prevalence (%)**	**No. of parasites**	***P* value**
	**≤1 (n=10)**	**1–2 (n=18)**	**>2 (n=23)**	***P* value**	**M (n=44)**	**F (n=7)**			
*Taenia taeniaeformis*	0	100	65.2	0.001	65.9	57.1	64.7	80	0.8
*Mesocestoides lineatus*	0	88.9	39.1	0.001	50	42.8	49.02	30	0.79
*Dipylidium caninum*	20	50	17.4	0.027	29.5	28.5	29.41	20	0.86
*Taenia hydatigena*	0	44.4	8.7	0.017	22.7	0	19.6	13	0.37
*Ancylostoma tubaeforme*	0	0	13	0.4	6.8	0	5.9	4	0.77
*Toxascaris leonina*	0	33.3	0	0.014	13.6	0	11.77	10	0.68
*Toxocara cati*	30	100	100	0.001	86.3	85.7	86.3	125	0.98
EPG	24±4	23.5±4.5	33.7±6.3	0.93	27.75±6.5	38.7±3.3	-	-	0.9
*Ctenocephalides felis*	20	38.9	60.7	0.05	50	42.9	80	160	0.05
*Ctenocephalides canis*	10	5.6	13.04	0.29	6.8	28.6	20	110	0.93

Notes: EPG: egg per gram of feces, F: female, M: male

The infection intensities were variable in different age groups, ranging from 24±4 to 33.7±6.3 eggs per gram of feces. There was significant correlation between infection intensities and age groups (χ^2^=4.425, df=2, *P*<0.05). The prevalence was significantly higher in the cats aged 1–2-year-old than the other age groups (χ^2^ =3.538, *df*=2, *P*<0.05). However, there was no significant difference in prevalence between male (EPG=27.75±9) and female (EPG=38.7±3.3) cats (χ^2^ = 2.366, df=1, *P*>0.05).

The identified species of helminths, their prevalence, and cross-infection rates are given in Tables 1 and 2. Laboratory identification indicated that the most widespread gastrointestinal parasitic helminths of the investigated cats were cestodes (four species) and nematodes (three species). Based on the necropsy methods, the highest percentage of helminth infection belonged to *Toxocara cati* (86.3%), followed by *Taenia taeniaeformis* (64.7%), *Mesocestoides lineatus* (49.02%), *Dipylidium caninum* (29.41%), *Taenia hydatigena* (19.6%), *Toxascaris T. leonina* (11.77%) and *Ancylostoma tubaeforme* (5.9%). *T. cati* (100% with a total of 125 helminths and 10.24±3.75 eggs per gram of feces) and *T. taeniaeformis* (100% with a total number of 80 helminths) were the most prevalent helminths in the young stray cats. Three cats (5.9%) harbored the adult helminths of at least one species. Among all the helminthes, only prevalence of *A. tubaeforme* was not significantly different among the different age groups (*P*>0.05). Of the all examined stray cats, 3 (5.9%) were cross infected with more than one helminth species. In most intensive case, two stray cats (3.9%) were cross infected with six helminth species (Table 2).

**Table 2:** Prevalence of the cross-infection with helminth species in the stray cats of Ahar City, Iran

**No. of helminth species**	**No. of infected cats**	**Prevalence (%)**
1	3	5.9
2	15	29.4
3	9	17.6
4	11	21.6
5	4	7.5
6	2	3.9

### Ectoparasites prevalence and diversity

Flea infestation was only detected in the examined cats with overall prevalence of 60.78% (31/51). The infestation was caused by two species of the genus *Ctenocephalides* (Siphonaptera: Pulicidae), i.e. *Ctenocephalides felis* and *C. canis*. The cat flea, *C. felis* was the most common flea infesting the cats in the age group of 2–3 yr old (80%, 41/51). There was a pruritis skin sign with no flea cross infestation in the examined stray cats. The flea index (average number of flea per infested animals) was recorded to be 5.29. There was no significant difference in flea prevalence between male and female cats (*P*>0.05) (Table 1).

## Discussion

Zoonotic infections are responsible for over 60% of all human infectious diseases worldwide (14). Some of the most important and well-known human zoonoses are caused by helminth parasites and are still prevalent in most regions of the world (15). Being in close contact with human communities, the stray cats are considered as a potential reservoir for some human diseases (16). They hunt infected intermediate or paratenic hosts like rodents in their life cycles, which act as important sources of helminth infection (17).

Information on the prevalence of endoparasites infection, in particular, helminths, is essential to implement effective control programs. The prevalence rates of helminth infection in the stray cats were comparable to those reported from different parts of Iran, i.e. Kashan (97.3%) (18), Bandar-e-Anzali (90%) (19), and Ahvaz (86.4%) (20). High prevalence of the parasites in stray cats was also reported from Thailand (94%) (11), Spain (89.7%) (21), Brazil (89.6%) (22) and India (85.2%) (17). On the other hand, there is a report of low infection rate from Iraq (38.75%) (23). The cats often live freely in urban and residential areas as a predator of rats. Their growing number with easy access to the intermediate hosts such as rodents, and defecating in public areas can be the main reasons for recording such a high parasitic prevalence in this study.

While prevalence of helminths was significantly higher in young stray cats, the presence of the helminths infection in different age groups of the cats indicates the susceptibility of all their life stages to the infection. This finding was in line with other reports elsewhere (24–26). Helminths infection rate was highest in the cats less than one-year-old in Thailand (11). These controversial results may be due to the differences in samples size, the frequencies of the cats in different age groups, availability of infected foods for different age classes or genetics and level of immunity against the helminths infection in the examined cats.

The prevalence of the infection with helminths was not significantly different between two sexes of the examined stray cats. Several researchers made a similar observation from different regions of Iran (18, 19, 24–25). The helminths infection was higher in female (95.5% versus 89.6% in males) stray cats (27). These differences may be due to ease in access of female or male cats to the intermediate host or the parasites, sample size of each sex, and the study season in association with reproductive period in females.

In Iran, *Toxocara* species are prevalent ascaridoid nematodes of cats causing zoonotic parasitic diseases (28). The present investigation elucidated that *T. cati* was the most predominant nematode species. The reported prevalence of *T. cati* in Iran are 67.5% (29) and 92.9% (27) in the cats of Tehran and Shiraz, respectively. Nevertheless, prevalence was much lower than those reported in the previous and recent decades from Iran, i.e. from Shiraz 26.7% and 42.6% (24,28), Bandar-e-Anzali 8% (19), Ahvaz 8.3% (30), Tehran 9.4% (31). However, the prevalence of the infection with *T. cati* in this study was nearly similar to the reports from Spain (55.2%) (21) and England (53.3%) (32). The intensity of infection was ranged 1–39 helminths with a mean of 10.25 (28). In another report, the average intensity was 6.52 with a range of 1–50 helminths per cat (24). The intensity was ranged 1–32 helminths with a mean of 7.3 (7). The mean intensity of *T. cati* was 3 in each stray cat from northern Iran (19). With regard to the sex of the helminths removed from the examined stray cats in this investigation, female nematodes were prevalent. Presence of the free-living stray cats and daily discharge of *Toxocara* eggs in the environment (about 200000 eggs/day) can be considered as a reason of this fact why ascaridoid nematodes of cats are prevalent (28).

In the present study, *T. taeniaeformis* was the second most frequent helminth species in the examined stray cats, while its prevalence was very low (0.95%) in the stray cats of Tehran, Iran (33). Recent epidemiological studies in Iran demonstrated the widespread prevalence of infection with *T. taeniaeformis* in the stray cats, i.e. 12.3% in Shiraz (27) and 2% in Bandar-e-Anzali (19). Borthakur and Mukharjee (17) reported that *T. taeniaeformis* was the most prevalent helminth (70.4%) in the Indian stray cats. Similarly, *T. taeniaeformis* had the highest prevalence in the stray cats of Qatar (34). In contrast, its low prevalence rates were reported in the stray cats of Nigeria (9.6%) (35) and the Netherlands (3%) (36). The optimal environmental and climate conditions such as suitable relative humidity and moderate temperatures, during the course of this study and also, presence of rodents as intermediate hosts plus low sanitary status, which are essential for stabilized life cycle of helminths, could be the causes of high prevalence of *T. taeniaeformis* in the stray cats of Ahar Municipality.

Fleas are one of the main causes of skin pruritus in outdoor cats and through transmitted variety of infectious agents and cause anemia in young cats or other animals (37). In current study, prevalence of the detected fleas in the cats was remarkable and slightly higher in the female’s aged 2-3 yr-old. Our observation resembles earlier studies (38, 39) and may be because female cats have longer life than the males (39).

*Centenocephalides felis* is one of the most frequent ectoparasites of the companion animals worldwide and is generally regarded as the prominent species found in cats (5, 25, 40). In the current survey, *C. felis* was also the predominant flea species in the infected cats. A reasonable explanation for such high infection in the cats can be that these animals freely roam to the resting places away from their permanent dens, where ectoparasites may persist (5). Low prevalence of *C. canis* in this study was in contrast with its prominence over *C. felis* in the infected cats from New Zealand (41), Greece (42) and Germany (5). This evidence suggests regional differences in the distribution of the ectoparasites rather than a tendency towards extinction as has been postulated due to other observations (43). Both of these flea species were isolated from the cats in several other countries including Mexico (44), Germany (5), UK (6) and Hungary (45).

The life cycle of these ectoparasites continues throughout the year (5). In a recent investigation, the coincidence of *C. felis* and *C. blakei* was reported in the stray cats of Mashad City, northeastern Iran (25).

## Conclusion

The stray cats, which are living in close contact with human and domestic livestock, can be highly polluted by several infectious agents and as a result, a potential source of various diseases. This needs to be considered from the viewpoint of zoonotic agent’s transmission and general public health impact. In this regard, effective parasites control and further studies for a better understanding of geographical and seasonal distribution of the parasites are necessary.
